# External and internal consistency of choices made in convex time budgets

**DOI:** 10.1007/s10683-016-9506-z

**Published:** 2017-01-06

**Authors:** Anujit Chakraborty, Evan M. Calford, Guidon Fenig, Yoram Halevy

**Affiliations:** 1 Vancouver School of Economics, University of British Columbia, 6000 Iona Drive, Vancouver, BC V6T 1L4 Canada; 2 Department of Economics, Krannert School of Management, Purdue University, West Lafayette, IN 47907 USA; 3 Department of Economics, University of Saskatchewan, Saskatoon, SK S7N 5A5 Canada

**Keywords:** Discounting, Time preference, Weak axiom of revealed preference, Choice from budget lines, Elicitation of preferences, MPL

## Abstract

We evaluate data on choices made from convex time budgets (CTB) in Andreoni and Sprenger (Am Econ Rev 102(7):3333–3356, 2012a) and Augenblick et al. (Q J Econ 130(3):1067–1115, 2015), two influential studies that proposed and applied this experimental technique. We use the weak axiom of revealed preference (WARP) to test for external consistency relative to pairwise choice, and demand, wealth and impatience monotonicity to test for internal consistency. We find that choices made by subjects in the original Andreoni and Sprenger (Am Econ Rev 102(7):3333–3356, 2012a) paper violate WARP frequently; violations of all three internal measures of monotonicity are concentrated in subjects who take advantage of the novel feature of CTB by making interior choices. Wealth monotonicity violations are more prevalent and pronounced than either demand or impatience monotonicity violations. We substantiate the importance of our desiderata of choice consistency in examining effort allocation choices made in Augenblick et al. (Q J Econ 130(3):1067–1115, 2015), where we find considerably more demand monotonicity violations, as well as many classical monotonicity violations which are associated with time inconsistent behavior. We believe that the frequency and magnitude of WARP and monotonicity violations found in the two studies pose important confounds for interpreting and structurally estimating choice patterns elicited through CTB. We encourage researchers employing CTB in present and future experiments to include consistency tests in their design and pre-estimation analysis.

## Introduction

Elicitation of time preferences in the discounted utility (DU) model requires simultaneous estimation of the felicity and discount functions. To demonstrate, consider a subject whose preferences over consumption streams are represented by a time-invariant^1^ DU model, and decides at time 0 on her consumption in periods $t$ and $t+k$. Her utility is given by $U\left( c_{t},c_{t+k}\right) =D\left( t\right) u\left( c_{t}\right) +D\left( t+k\right) u\left( c_{t+k}\right)$, where $D\left( \cdot \right)$ is the subject’s discount function, and $u\left( \cdot \right)$ is her felicity function. Estimation of a discount function that is based on indifference between consumption of $c^{1}$ at time $t$ and $c^{2}\left(>c^{1}\right)$ at time $t+k$ (and nothing in the other period), e.g. through multiple price list (MPL), implies that $D\left( t+k\right) /D\left( t\right) =u\left( c^{1}\right) /u\left( c^{2}\right)$. It is well known that if the researcher assumes linear $u\left( \cdot \right)$ while the true felicity function is concave, it will bias the estimated $D\left( t+k\right) /D\left( t\right)$ downwards.^2^

To cope with this difficulty Andersen et al. (2008) used the fact that under the standard Discounted Expected Utility model risk and time preferences are intimately linked: a concave utility function exhibits both atemporal risk aversion and a desire for intertemporal smoothing of consumption. Their double multiple price list (MPL) procedure uses one atemporal multiple price list to estimate risk preferences and a second intertemporal multiple price list to estimate time preferences. They use the curvature of the atemporal utility function in order to adjust the estimation of the discount function.

Andreoni and Sprenger (2012a, abbreviated exchangeably as AS in the following) proposed an interesting alternative according to which a single instrument can be used to jointly estimate the felicity and discount functions, without explicitly relying on the subject’s risk preferences. Andreoni and Sprenger’s convex time budgets (CTB) are a convexification of pairwise choices made on lines in the intertemporal MPL and allow the economist to directly measure intertemporal substitution. In their design the subject faces linear experimental budgets, which allow her to choose interior allocations between payments at two time periods ($t$ and $t+k$). One can rationalize such interior allocations if the subject’s preferences between $c_{t}$ and $c_{t+k}$ are (weakly) convex. It thus provides a way to directly adjust the measurement of the subject’s discount function for intertemporal substitution without the need to explicitly invoke expected utility.^3^ Andreoni and Sprenger (2012a), and their closely related study (Andreoni and Sprenger 2012b), have been followed by a large number of applications and comments.^4^

The current paper provides commentary and guidance for economists who wish to use CTB to measure time preferences. Specifically, we discuss a methodology for measuring the consistency of subject-level choices with a very general model of intertemporal choice (more general than the DU model). A key element of this methodology requires the inclusion of two convex budgets that differ only in their income level in the CTB design, which makes a direct test of wealth monotonicity possible.^5^ We illustrate our approach using the data set of AS (on time allocation of money) and on the most influential application of CTB to date—the work of Augenblick et al. (2015), which investigates allocation of effort over time.

In the AS study, we find surprisingly high rates of violations of the general model of intertemporal choice that we consider. For the subjects who did not exhibit any curvature in their CTB choices, we directly estimate their discount factor based on the three choice lists and the corresponding CTBs *assuming* linearity of the felicity function, for the sake of comparison. We find WARP violations between choices made on CTB and choice lists for these subjects, and most of these violations are in the direction of exhibiting lower impatience in CTB than in choice lists. This could be an explanation for why Andreoni and Sprenger (2012a) obtain reasonably high CTB discount factors for these subjects even though their discount factors are not adjusted upward (as there is no evidence that their felicity function is concave). In the Augenblick et al. (2015) paper we find substantial rates of demand monotonicity violations, especially in their replication study. The latter violations are accompanied by violations of classical monotonicity, which in turn are empirically associated with time inconsistent behavior. Choices that violate classical monotonicity cannot be rationalized by a monotone utility function, a fact that relates this finding to the literature on “decision-making quality” (Choi et al. 2014)—if rationalizability of choices by a utility function is a marker of choice quality, then there is definitely some relation between the decision making quality and adherence to the normative standard of time consistency. We believe these surprising findings highlight the importance of implementing our suggested methodology before and after using CTB data for estimation.

In what follows, we suggest possible behavioral mechanisms (for example, magnitude effect, reference dependence, subject confusion, experimental design) that may generate the observed inconsistencies. We hope that our work encourages future research to further address these questions.

### Consistency requirements and summary of results

We identify three basic properties that allocations in a CTB design should satisfy in order to be rationalizable by a very general model of intertemporal choice: allocations should satisfy *wealth monotonicity* (*normality*) implying that $c_{t}$ and $c_{t+k}$ should be weakly increasing in wealth, holding interest rate constant; $c_{t}$ should be weakly decreasing in interest rate (*demand monotonicity*), holding the dates $t$ and $t+k$ and wealth normalized to the later date constant, with $c_{t}$ strictly decreasing whenever $(c_{t},c_{t+k})$ is interior; allocations should be consistent with *impatience* implying that as the later (earlier) date is shifted away from the present, $c_{t}$ should weakly increase (decrease), holding the earlier (later) date, price ratio and wealth constant. Additionally, we use the fact that AS also included some multiple price lists in their design to test for violations of the *weak axiom of revealed preferences* (*WARP*). The various monotonicity criteria for which we evaluate the empirical demand should not be confused with monotonicity of the utility function with respect to $\left( c_{t},c_{t+k}\right) .$ In particular, wealth and demand monotonicity are consequences of the very weak assumption that $c_{t}$ and $c_{t+k}$ are normal goods. When choices are inconsistent with monotonicity of the utility function we say that they violate “classical monotonicity.”

We document the level of adherence of choices (at the individual level) to the above very mild external and internal consistency requirements. We find a very high level of WARP violations among the many subjects who made corner choices in Andreoni and Sprenger (2012a). Violations of all three internal measures of monotonicity are concentrated in subjects who make interior choices and thereby take advantage of the novel feature of Andreoni and Sprenger’s CTB experimental design. Wealth monotonicity violations are more prevalent and pronounced than either demand or impatience monotonicity violations (except when all choices are interior).

We then investigate the consistency of choices in the Augenblick et al. (2015) study. This is the most significant application of CTB to date, as it tries to distinguish discounting of primary rewards (or costs—implemented through an effort task) from discounting of monetary rewards (as in the majority of experimental studies of intertemporal preferences). One of the important findings of Augenblick et al. (2015) is that subjects tend to make interior choices much more often when deciding on allocation of effort than of money, and that there is significantly more time inconsistency (in the form of present bias) in effort. Augenblick et al. (2015) includes two experiments: the design of the first experiment may confound present bias with other sources of time inconsistency,^6^ and the second experiment was designed in order to eliminate some of these potential confounds. Although Augenblick et al. (2015) did not include some of the important comparative static treatments that were part of Andreoni and Sprenger (2012a), we are still able to test for demand monotonicity. In the first experiment we find levels of demand non-monotonicity in effort that are comparable to interior choices (on the allocation of money) made in AS. However, in the replication study we find higher levels of demand monotonicity violations, that we could not account for even after taking into account rounding effects that allowed subjects to make choices that are inconsistent with monotone preferences and a higher number of interest rates faced by subjects. Additionally, we find that non-adherence to classical monotonicity is significantly associated with time inconsistent choices.

We believe that the findings reported here motivate the following fundamental question: are choices made in CTB reflective of deep and stable preferences? We urge researchers to study the source of the documented inconsistent behavior in order to decide if it could be attributed to the implementation of CTB in the two studies we cover or if it reflects some behavior that the standard discounted utility models (and hence the structural estimation methods used in the mentioned studies) are not equipped to handle. We are of the opinion that inclusion of the wealth shifter in Andreoni and Sprenger (2012a) was a crucial design innovation, and we recommend that future CTB papers include a similar ‘wealth shifter’ to facilitate analysis. At a minimum, we would encourage future researchers to test their CTB data for consistency with the internal measures of monotonicity before applying the data in new settings or using it for the purpose of structural estimation.

We proceed as follows. Section 2 provides a brief overview of the extremely active literature on measuring time preferences. Section 3 discusses Andreoni and Sprenger (2012a) in detail; we first describe how to identify wealth monotonicity, demand monotonicity, impatience and WARP violations in the AS dataset, and then present the results of our investigation. Section 4 provides a similar analysis of Augenblick et al. (2015). Section 5 concludes.

## Literature review

Andreoni and Sprenger (2012a, b) have been among some of the most influential experimental papers in recent years, generating a significant amount of academic discussion about the experimental methodologies of estimating and understanding risk and time preferences. The most significant contribution of Andreoni and Sprenger (2012a) is the parsimony of the CTB framework for estimating time preferences without explicitly relying on expected utility in order to adjust the discount function for the curvature of the felicity function. The authors also do a very convincing and careful job of equalizing the subject convenience and confidence for present and future payments to measure present bias separately from the confounds of differential transaction costs. Andreoni and Sprenger (2012a) find very little evidence for present bias and curvature in the atemporal felicity function. The closely related paper Andreoni and Sprenger (2012b) compares CTB decisions in which payments on both dates are certain, to ones in which payments are risky, as realized by two independent lotteries. The authors hypothesise that subjects’ choices are governed by an (atemporal) utility function that is more concave than the one employed under conditions of certainty, as subjects choose more balanced portfolios of sooner and later payments under the risky condition.

Andreoni and Sprenger (2012a, b) led a host of follow-up studies which have used this methodology for estimation purposes. CTB has been otherwise used to study the evidence for present bias among particular sectors of the population (e.g. Giné et al. 2016; Ashton 2014; Kuhn et al. 2014; Carvalho et al. 2016). Aside from being a huge influence on the experimental literature, Andreoni and Sprenger (2012b) has also generated a range of comments on the robustness and interpretation of its findings. Cheung (2015) investigates the robustness of AS (2012b) to alternative experimental design. In a particularly interesting translation of the key AS (2012b) CTB treatment into a double MPL environment, Cheung uses an MPL where payments on both dates are received with 50% probability, and contrary to AS (2012b) he finds “very little evidence of difference of any systematic deviation in discounting behavior under risk as compared to certainty.” Cheung also finds evidence for non-linearity in intertemporal preferences when, in the absence of diversification opportunities (the risks across time being correlated), the proportion of interior allocations falls between those of no risk and independent risks. Miao and Zhong (2015) utilize two additional CTB risk treatments (one of them similar to that of Cheung) to show that the behavior exhibited in temporal risk environments is more consistent with a model which separates risk attitudes and intertemporal substitution (like Epstein and Zin 1989; Halevy 2008) than the one suggested in AS (2012b). Epper and Fehr-Duda (2015) demonstrate that probability weighting in rank-dependent utility models that take their entire temporal portfolios into account are able to explain subjects’ preference for intertemporal diversification as well as their proneness to intertemporal common-ratio violations and, therefore, all the major AS findings. Schmidt (2014) offers a different perspective: if the monetary payments in AS (2012a, b) are interpreted as income instead of as consumption, then arbitrage and portfolio risk minimization in a DEU framework could justify why subjects choose more interior solutions in the correlated temporal risk task than in the deterministic temporal task.

Harrison et al. (2013) is another critical comment that directly addresses Andreoni and Sprenger (2012a). One of the key arguments of Harrison et al. (2013) is that the large number of corner choices in the CTB data generates a bi-modal data set which is not well suited to analysis via non-linear least squares estimation techniques. The authors note that more appropriate econometric techniques, which attempt to match the full distribution of the data, imply that the data is best rationalized with a convex utility function. Furthermore, they argue that convex utility functions are *a priori* implausible in this environment and they therefore question the quality of the data.

Augenblick et al. (2015) has been the most successful behavioral application of the CTB design. The authors use CTB to show present bias while using primary rewards (effort tasks). For sake of comparison, they pair this effort study with a companion monetary discounting study and find very limited time inconsistency in monetary choices. We analyze in greater detail demand monotonicity violations in the effort domain in Sect. 4.

## Andreoni and Sprenger (2012)

The Andreoni and Sprenger (2012a) design includes nine choicesets per subject, where each choiceset is a collection of five CTB tasks between payments at $t$ and at $t+k$ (where $t=0,7,35$ and $k=35,70,98$ measured in days). Eight out of the nine choicesets contain a wealth shift which could be used to test for wealth monotonicity. Demand monotonicity is tested by the other four CTB tasks within a choiceset. Impatience is tested by comparing across choicesets belonging to the same subject. When evaluating wealth monotonicity we allow for the non-generic possibility of linear preferences with marginal rate of substitution between $c_{t}$ and $c_{t+k}$ equal to the gross interest rate over *k* days in which the wealth shift occurs, i.e. $1+r=1.25$. In this case, the demand is a correspondence and wealth monotonicity as defined above need not hold.^7^

AS included three choice lists (MPL) that correspond to three choicesets. Each one of these choice lists included four pairwise choices that corresponded to CTB. In other words, on these lines of the choice list a subject was asked to make a pairwise choice between the two points in which each CTB intersects the horizontal axis ($c_{t+k}=0$) and the vertical axis ($c_{t}=0$). In the CTB task the menu of allocations the subject was allowed to choose from included these two allocations *and* all interior allocations. We use this set-up to test for violations of the Weak Axiom of Revealed Preference (WARP), which requires that if an alternative is chosen from a menu and is available in a sub-menu then it should be chosen from the sub-menu as well. If in the pairwise choice a subject chooses one corner while in the CTB she chooses the opposite corner this contradicts WARP. The implication is that there exists no complete and transitive preference that can rationalize these choices.

### Corner choices

Although the CTB design allowed for interior choices, 70% of all choices were made at the corners of the budget set. 36 of the 97 subjects made *only* corner choices. There is little within subject variation and between subject heterogeneity among these subjects. Nineteen of these subjects had the exact same choice sequence for all tasks: they chose the later-larger reward whenever the “gross interest rate” was greater than 1. Four other subjects chose the later-larger reward for all 45 CTB tasks, irrespective of interest rate and time horizon.

### WARP violations

Out of the 36 subjects who made all corner choices in CTB, we found 43 violations of WARP.^8^ This is especially impressive if one considers that 17 of them always chose later consumption in the CTB and switched immediately in the choice lists (always chose later consumption). Therefore WARP violations could be detected only among the remaining 19 subjects. The direction of WARP violations is not random: 34 violations are in the direction of exhibiting less impatience in CTB than in choice list, while only 9 are in the opposite direction.

**Fig. 1:**
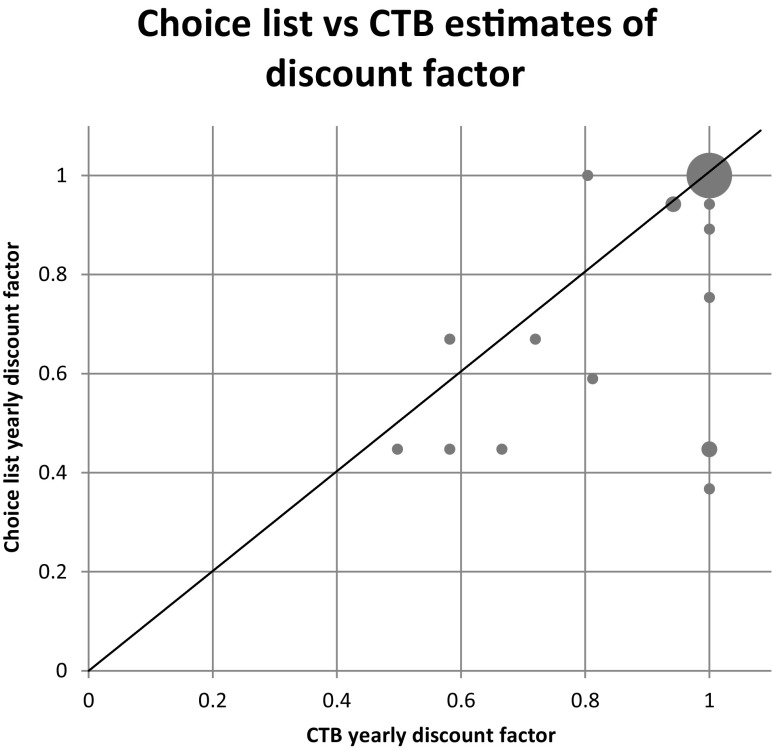
Choice list versus CTB estimates of discount factor for the 36 all-corner subjects

Since these subjects did not exhibit any curvature in their CTB choices, we can directly estimate their discount factor based on the three choice lists and the corresponding CTBs *assuming* linearity of the felicity function. One should not adjust for curvature for these subjects, since their intertemporal decisions did not suggest any concavity of the felicity function.^9^

The results are plotted in Fig. 1.^10^ We find that for 11 subjects the discount factor estimated from CTB data would be higher than the one estimated from choice list data, while for two subjects the relation between the discount factors would be in the opposite direction. Note that the choices made by the 17 subjects who always chose later consumption can be rationalized with a discount factor of 1, and one cannot form a point estimate of the discount factors of 4 other subjects who always chose immediate consumption in at least one of the three CTBs.^11^

Among the other 61 subjects who made at least a single interior choice in the 45 CTB tasks we find a similar directional effect of WARP violations. If one of the three choicesets that has a comparable choice list has all corner choices, we find 23 WARP violations in the direction of exhibiting lower impatience in CTB than in choice list and none in the opposite direction. In choicesets with interior CTB choices (where the potential to observe direct WARP violation is smaller) we found ten violations in the direction of exhibiting lower impatience in CTB than in choice list and five in the opposite direction. It is important to note that owing to the WARP violations, there is no model of complete and transitive preferences that could potentially help us understand the correlation between impatience parameters estimated via CTB and MPL (DMPL) techniques within or across studies.

The WARP violations indicate an inconsistency between choices elicited via CTB and choices elicited via a choice list, and the strong correlation between WARP violations and impatience measures suggest that the inconsistency is, in some sense, structural rather than random. One possible interpretation of the WARP violations [following Ok et al. (2015)] is that CTB induces a form of reference dependence by moving away from binary choices.^12^ However, it could be argued that a choice list may also be susceptible to reference dependence—consider for example a list starting from low interest rates compared to a list starting from high interest rates [see Andersen et al. (2006) for further discussion].

While the presence of inconsistency between the choices in the CTB and MPL tasks does not necessarily privilege either elicitation procedure, the results are nevertheless both disconcerting and very interesting. We believe that understanding the nature of the discrepancies between the two elicitation mechanisms is an important open question.

### Demand and wealth monotonicity

As the 36 subjects with all corner choices did not take advantage of the convexification offered by the CTB, we believe it would be misleading to include them in evaluating CTB for internal consistency (monotonicity). Hence, the analysis below concentrates on the remaining 61 subjects with at least one interior choice.

#### Frequency

Table 1 reports the frequency of choicesets that have wealth or demand monotonicity violations as a function of the number of interior choices made in a choiceset.

**Table 1:** Demand and wealth monotonicity violations as a function of number of interior choices

# of interior choices in a choiceset	# of choicesets	# of choicesets that exhibit demand monotonicity violations	# of choicesets that exhibit wealth monotonicity violations	# of choicesets that exhibit either wealth or demand monotonicity violations
0	435^a^	1	9	10
1	101	10	26	34
2	78	5	31	34
3	80	6	47	48
4	63	6	47	47
5	116	42	56	76
Total	873	70	216	249

^a^324 out of the 435 choicesets with no interior choice (almost 75%) belong to the 36 subjects with only corner solutions

The frequency of demand monotonicity violations is below 10% for choicesets that contain 4 or fewer interior choices. However, more than 36% of choicesets with all interior choices have demand monotonicity violations. The frequency of wealth monotonicity violations is considerably higher: around half of the choicesets with at least one interior choice have a wealth monotonicity violation.

**Table 2:** Joint frequency of number of interior choicesets (by subjects) and number of interior choicesets that do not violate (demand and wealth) monotonicity (by subject), restricted to subjects who have at least one interior choiceset

# of interior^a^	# of monotone interior^a^ choicesets										Total
choicesets	0	1	2	3	4	5	6	7	8	9	
1	**0**	2									2
2	**1**	**0**	0								1
3	**2**	**0**	0	2							4
4	**0**	**2**	**0**	0	1						3
5	**1**	**1**	**0**	0	2	1					5
6	**0**	**2**	**0**	**0**	0	1	1				4
7	**0**	**0**	**1**	**1**	0	0	0	2			4
8	**0**	**3**	**2**	**0**	**0**	2	1	1	0		9
9	**1**	**8**	**5**	**4**	**2**	0	2	2	0	5	29
Total	5	18	8	7	5	4	4	5	0	5	61

The bolded entries highlights subjects that had violations in half or more of their interior choicesets

^a^A choiceset is considered “interior” if at least a single choice (out of 5) is not at the corners of the budget line

Table 2 reports, for the 61 subjects with at least one interior choiceset, the distribution of subjects satisfying wealth and demand monotonicity as a function of the number of interior choicesets. A choiceset is considered interior if at least a single choice (out of five) is not at the corners of the budget line ($c_{t},c_{t+k}>0$).^13^ Table 2 reveals that more than half of the 61 subjects violate monotonicity in at least half of their interior choicesets (the bolded entries in the table).

#### Magnitude

The two tables above demonstrate the high frequency of non-monotone choices in interior choicesets, especially as a response to wealth changes. We now turn to measure the magnitude of these behaviors. We calculate the magnitude of a wealth monotonicity violation by the number of tokens required to be reallocated in order to eliminate the violation at the higher wealth level. Our wealth monotonicity measure differs substantially from that reported in footnote 25 by Andreoni and Sprenger (2012a). We find that there are 216 violations of wealth monotonicity, with an average size of 24.46 tokens, which is 24.46% of the experimental budget or $4.89 of $$c_{t}$$ at the higher wealth level.^14^ That is, conditional on violating wealth monotonicity, the magnitude of the measure is almost as high as the equivalent measure calculated for random choice: if choices are generated at random using a uniform distribution over the tokens allocated to $$c_{t}$$, independently among the two budget lines, the expected value of our measure would be approximately 27 tokens.^15^ Andreoni and Sprenger report an average adjustment of just 1.67 tokens to restore wealth monotonicity. There are three differences between our calculation procedure and the one used in AS (presented in decreasing order of importance).

First, we take the absolute value of violations. AS mistakenly defined non-monotonicity that is expressed as an over-allocation to $c_{t+k}$ (and under-allocation to $c_{t}$) as a negative number, while non-monotonicity that is expressed as an under-allocation to $c_{t+k}$ (and over-allocation to $c_{t}$) as a positive number. Because both over- and under-allocation to $c_{t+k}$ are prevalent across the population, violations cancel out at the aggregate level. Taking the absolute value of the violations accounts for 24% of the total discrepancy: starting from the 1.67 tokens reported in AS, correcting for this increases the measure to 7.03 tokens. Second, accounting for almost all of the residual discrepancy, we include only choicesets with a wealth monotonicity violation in the denominator. In contrast, AS use a denominator that includes all choicesets with a wealth shift, rather than just choicesets with a wealth monotonicity violation. We believe that the AS approach, by including the 36 subjects who made only corner choices (and had no wealth monotonicity violation), artificially dilutes the magnitude of monotonicity violations performed by subjects who responded to the convexification offered by the CTB design by making interior choices. Of course, this is mostly an accounting decision, and hence, we consider it less important than our first point of departure. Lastly, we measure violations using whole numbers of tokens, thereby reflecting the choice environment presented to subjects, accounting for less than .5% of the total discrepancy. AS use integer number of tokens when calculating the magnitude of demand monotonicity violations, but not when calculating the magnitude of wealth monotonicity violations.

Turning now to demand monotonicity, we calculate the magnitude of demand monotonicity violations by finding the minimal amount of $$c_{t}$$ that needs to be reallocated per choiceset to restore monotonicity. There are 70 choicesets with demand monotonicity violations, with an average size of 17.4 tokens and a value (at time $$t$$) of $3.02.^16^

Another measure of the degree of non-monotonicity within a choiceset is to calculate the smallest number of choices that must be removed from a choiceset to restore monotonicity.^17^ For the 249 choicesets that exhibit at least one non-monotonicity, the average number of data points that must be removed is 1.2. This figure includes the 179 choicesets that exhibit only wealth non-monotonicity and therefore require the removal of only a single data point; for the 70 choicesets that exhibit demand non-monotonicity the average number of data points that must be removed is 1.8.

There is a possibility that the income non-monotonicity we identify is an outcome of subjects exhibiting different temporal preferences for different stakes, known in the literature as the magnitude effect (see Thaler 1981; Frederick et al. 2002). In studies that vary the outcome sizes, subjects appear to exhibit greater patience toward larger rewards. There are 152 instances of wealth monotonicity violations consistent with the subjects exhibiting greater patience, and 64 instances of the same in the opposite direction. The average size of reallocation required to restore monotonicity is 19.77 and 30.90 tokens, respectively. As a result, we suspect that the magnitude effect by itself is not sufficient to explain the frequency and magnitude of wealth monotonicity violations resulting from miniscule changes in budget wealth. In any case, whether “magnitude effect” is the correct interpretation of this phenomenon is an open question that future research would hopefully shed light on. One step in that direction is the recent work of Sun and Potters (2016), which reports a significant “magnitude effect” in CTB tasks.

### Impatience monotonicity

Turning to impatience, there are 10 pairs of choicesets across which either $t$ is constant and *k* varies, or $t+k$ is constant and $t$ varies; these are the only pairs of choicesets in which it is possible to test for impatience. In a comparable pair of choicesets (in the sense described above), we test for impatience monotonicity as described in Sect. 1.1 for all pairs of choice tasks (one in each choiceset) with the same prices.

We find that 47 of the 97 subjects satisfy the impatience criterion for all 10 pairs of choicesets; restricting the sample to the 61 subjects with at least one interior choice, we find that only 12 subjects made choices consistent with impatience monotonicity, and that 17 subjects violate impatience monotonicity in at least 5 of the 10 choiceset comparisons.

### Monotonicity index

Finally, we calculate an index that measures the (approximate) minimal number of data points that need to be eliminated from an individual’s dataset in order to be consistent with the three monotonicity requirements. This index is close in spirit to the Houtman–Maks (1985) index which is used to calculate the maximal set of observations in a dataset that is consistent with the generalized axiom of revealed preference (GARP).^18^ Out of the 36 subjects with no interior choice, 35 subjects satisfy all monotonicity measures.^19^ Out of the 61 subjects with at least a single interior choice, in 22 datasets we need to remove four or fewer choices,^20^ in 21 datasets we need to remove between five to nine choices (more than 10% of choices) and in an additional 18 datasets one needs to remove 10 or more choices (more than 20% of the total number of choices).

## Augenblick et al. (2015)

One critique that can be levelled against measuring time preferences using monetary payments, as in AS (2012a), is that subjects’ responses may be driven by their access to credit and savings instruments rather than their underlying time preferences over consumption bundles. Augenblick et al. (2015) build on this argument, and compare the preferences elicited through CTB design using both monetary payments and effort tasks, where the effort tasks are possibly non-fungible and assumed to impose a dis-utility on the subject and therefore allow a direct measurement of time preferences with respect to the work-leisure trade off. In their first study (henceforth original experiment) subjects allocate both cash and units of effort over two dates, using a within-subject design. They also run a second study (henceforth replication experiment) in which they implement a between-subject design to replicate the findings of the first study. Augenblick et al. (2015) identify two key differences between the monetary tasks and the effort tasks. First, present bias is found only in the effort domain and, second, the proportion of interior choices is much higher in the effort domain. This result supports the critique of monetary tasks as a tool for measuring impatience due to the fungibility of money.

In the following subsections we analyze the rate of violations of our behavioral desiderata across both the monetary and effort tasks. We believe it is a useful exercise for three reasons. First, Augenblick et al. (2015) has been the most influential application of CTB on “primary versus monetary rewards”, a topic that is contested between some behavioral and experimental economists. Second, the interface used in the Augenblick et al. (2015) is different from the one used in AS (2012a), so it allows us to evaluate if the demand monotonicity we documented in the latter is a consequence of interface subjects faced in the original AS study. Third, one key implication of our data analysis for the design of future CTB experiments revolves around the divisibility of effort tasks, and we will discuss this issue in detail in Sect. 4.1.1.

### Data Analysis

**Table 3:** Original study, work data

# of interior	# of demand					Total
choices	monotonicity violations					
	0	1	2	3	4	
0	77	0	0	0	0	77
1	150	0	1	0	0	151
2	15	8	1	0	0	24
3	26	15	4	0	0	45
4	28	12	2	0	2	44
5	224	62	51	22	20	379
Total	520	97	59	22	22	720

Number of interior choices (rows) crossed with number of demand monotonicity violations (cols)

**Table 4:** Original study, money data

# of interior	# of demand			
choices	monotonicity violations			Total
	0	1	2	
0	289	0	0	289
1	35	2	0	37
2	9	0	0	9
3	5	7	1	13
4	3	2	2	7
5	23	5	2	30
Total	364	16	5	385

Number of interior choices (rows) crossed with number of demand monotonicity violations (cols)

Because the Augenblick et al. (2015) design does not include a wealth shift, we are not able to test their data for wealth monotonicity. Instead, we calculate the rate of demand monotonicity violations for the effort tasks in the original study and find the frequency of violations to be higher than the rate of violations in AS (27.8% compared to 8.0%).^21^ $$^{,}$$ 22 As in AS, the rate of violations is higher when all choices are interior (Table 3), 40.9% of choicesets with all interior choices have demand monotonicity violations (the corresponding proportion in AS is 36.2%) Table 4 displays the number of demand monotonicity violations in the monetary allocations; the rate of violations is rather low, as might be expected given that almost all choices are corner choices. For impatience monotonicity, there is very little evidence of violations for both effort tasks and monetary choices in the original study, and there was no scope for impatience monotonicity violations in the replication study.

#### Rounding of choices and classical monotonicity violations

One key aspect to consider when implementing CTB over effort tasks rather than over monetary rewards is the divisibility of the units. Because the effort tasks in Augenblick et al. (2015) are discrete, there is a complication in offering finely distributed discrete choices on a budget line. Augenblick et al. (2015) deal with this in two different ways: in the original experiment they vary the possible work allocations in the nearer period on a regular integer grid (presented to subjects as a slider), and then round-down the corresponding values of the other farther period choice using the budget equation.^23^ This rounding method can, for some interest rates, lead to the availability of dominated allocations. For example, when the task rate is 1.5, and $e_{t}$ may be chosen to lie between 0 and 50, then the allocations $(e_{t},e_{t+k})=(2,32)$ and $(e_{t},e_{t+k})=(1,32)$ are both available.

In the replication, the authors considered all possible pairs of earlier and later effort choices that would be on the budget line, and then rounded both of them (independently) to the nearest integer. This rounding method can create situations in which for a given “task rate” subjects are offered allocations which are below or above the budget line. For example $(e_{t},e_{t+k})=(41,17),\,(40,17)\,{\text{and}}\,(40,18)$ appeared as possible choices (and each was chosen by at least some subjects) for the same budget line.^24^ This implies that certain subjects chose allocations that are strictly dominated by other available allocations. We identify such choices as violations of *classical monotonicity*. The rate of such violations is alarmingly high in the effort treatment of the replication data: 62 of the 95 subjects selected 8 or more (out of a maximum possible 18) dominated allocations. Because of the nature of the slider interface presented to subjects we think that subjects were probably simply unaware that dominating choices were available.

**Table 5:** Replication study, work data

# of interior choices	# of demand monotonicity violations									Total
	0	1	2	3	4	5	6	7	8	
0	11	1	0	0	0	0	0	0	0	12
1	9	0	0	0	0	0	0	0	0	9
3	1	1	0	0	0	0	0	0	0	2
5	0	1	1	1	1	0	0	0	0	4
6	0	0	1	0	0	0	0	0	0	1
7	5	2	2	1	0	0	0	1	0	11
8	7	5	1	1	1	0	1	1	0	17
9	12	25	43	26	12	6	2	1	7	134
Total	45	35	48	29	14	6	3	3	7	190

Number of interior choices (rows) crossed with number of demand monotonicity violations (cols)

**Table 6:** Replication study, work data

# of interior choices	# of demand monotonicity violations								Total
	0	1	2	3	4	5	7	8	
0	11	1	0	0	0	0	0	0	12
1	9	0	0	0	0	0	0	0	9
3	2	0	0	0	0	0	0	0	2
5	0	1	3	0	0	0	0	0	4
6	0	1	0	0	0	0	0	0	1
7	5	4	0	1	0	0	1	0	11
8	7	6	2	0	0	1	1	0	17
9	37	34	29	15	11	3	0	5	134
Total	71	47	34	16	11	4	2	5	190

Number of interior choices (rows) crossed with number of demand monotonicity violations (cols) after “correction” for classical monotonicity violations

The frequency of demand monotonicity violations is also high in the effort treatment of the replication: only 45 out of the 190 total choicesets have no demand monotonicity violations, which is a failure rate of 76.3%. Table 5 shows the number of demand monotonicity violations for the effort tasks in the replication study. We recognize that this high frequency of demand monotonicity violations might have been due to subjects choosing “above budget-line” and “below budget-line” allocations on different offered budget sets, and that these choices may have been driven by an unawareness of other nearby feasible allocations. For each observation which would constitute a demand monotonicity violation along with a choice at the adjacent lower discount rate, we first note if the higher, later-period effort choice at the higher discount rate was above the budget line due to experimental design. In this case, we “correct” the higher later period choice by moving it onto the budget line (thus decreasing it), in an attempt to “remove” the demand monotonicity violation. Similarly, for observations related to demand monotonicity violations, we note if the lower later period effort at a lower discount rate was below the budget line. As before, we “correct” this lower later period choice by moving it on the budget line (thus increasing it), in an attempt to “remove” the demand monotonicity violation. Using this modified data set, in Table 6, we report a more conservative frequency of demand monotonicity violations. The frequency of violations is still quite high (Table 6) as close to 63% of adjusted choicesets exhibit demand monotonicity violation.

Another reasonable hypothesis is that higher frequency of failing demand monotonicity in the replication experiment could be due to the fact that there are 9 discount rates rather than 5 (as in AS and the original experiment of Augenblick et al.). One could select 5 of the 9 discount rates, and evaluate demand monotonicity on that smaller set of choices for comparison.^25^ The demand monotonicity violation rates in the reduced exercise are still high: 74 (before aforementioned “correction”) or 67 (after “correction”) choicesets out of 190 choicesets (38.9 or 35.3%) have demand monotonicity violations. For comparison, in the original experiment the frequency was 200/720 (27.8%). Our conclusion is that the higher failure rate cannot be attributed solely to the higher number of interest rates.

We think that the high frequency of classical monotonicity violations and demand monotonicity violations, particularly the former, point out that certain participating subjects were not always well versed with the choice environment, thus failing to recognize and consider strictly better choices. Our suggestion to future studies planning to implement CTB effort tasks, is to either use divisible effort tasks or else follow the truncation of choices used by Augenblick et al. (2015) in their original effort task experiment.

#### Classical monotonicity violation and time preferences

Finally, we touch on the question if there is a correlation between dynamically inconsistent choices and classical monotonicity violations. Given that estimation of present bias or lack thereof is one of the primary goals of Augenblick et al. (2015), we think the relation between the frequency of violations and observed temporal preferences is of primary importance. We use the following non-parametric method to identify time consistent choices. Each subject makes 2 decisions of $(e_{t},e_{t+k})$ for every discount rate, once at $t=0$ and again at $t>0$. For every discount rate, if the subject allocates the same amount of effort at date $t$ in both her choices ($|e_{t}^{0}-e_{t}^{t}|\le 1$, i.e, allowing a tolerance of 1), we identify that pair as time-consistent, otherwise we label the pair as dynamically inconsistent. Given that subjects make such pairs of choices for 9 different discount rates, the subjects can have 0–9 total pairs of time-consistent choices. In Table 7 we tabulate this number against the number of classical monotonicity violations.^26^ We find that time inconsistency is associated with classical monotonicity violations, so subjects with fewer dynamically inconsistent choice pairs make fewer classical monotonicity violations than the ones who have more.

The association between failure of classical monotonicity and time-inconsistency is an interesting empirical relation that provides some additional insight into the Augenblick et al. (2015) choice environment. Abiding by classical monotonicity is a marker of “decision-making quality”—choices that can be rationalized by an increasing utility function (Choi et al. 2014). It follows that there is a relation between the decision making quality and adherence to the normative standard of time consistency. Moreover, “low-quality” decisions that are associated with time inconsistent choices cannot be rationalized by any utility function, let alone by quasi-hyperbolic discounting one.^27^ As before, with violations of income monotonicity and WARP, we think this is a fascinating topic worthy of independent future study.

**Table 7:** Replication study, work data

# time- inconsistent choices	# of classical monotonicity violations		Total
	0–7	8–16	
0–3	12	12	24
4–6	13	13	26
7–9	8	37	45
Total	33	62	95

Number of time inconsistent choices (rows) crossed with number of classical monotonicity violations (cols). A Fisher’s exact test rejects the null hypothesis of independence at standard significance levels (p $=$ 0.04), suggesting a positive association between dynamic inconsistency and classical monotonicity violations

## Conclusion

Andreoni and Sprenger’s proposal to use CTB in order to measure time preferences represents a potentially important methodological advance. In principle, assuming discounted utility, such a method can allow a researcher to calculate a more precise measurement of the discount function by controlling for intertemporal substitution, without explicitly relying on expected utility. However, our examination of data gathered by Andreoni and Sprenger (2012a) and Augenblick et al. (2015) using this method uncovers some issues that need addressing.

Subjects who made only corner choices in CTB violate WARP very frequently relative to the pairwise choice benchmark. This hints at choices being dependent on the particular elicitation method and allows a relatively pessimistic interpretation that at least one of the following, corner choices in CTB or MPL choices cannot be interpreted as reflecting reasoned behavior or deep preferences. As a whole, the bias of WARP violations relative to the pairwise choice benchmark is in the direction of lower impatience (higher discount factor). Subjects with interior monetary choices are broadly consistent with demand monotonicity (except when all choices are interior) and the evidence for impatience monotonicity violations is moderate. However, the high frequency and substantial magnitude of wealth monotonicity violations in this data suggest that interior choices made in CTB (responding to the convexification) may be incompatible with standard stable preferences.^28^

The Augenblick et al. (2015) study does not include some of the experimental comparative-static controls from the AS (2012a) paper. In their original experiment, we find that the rate of demand monotonicity violation in the effort task is comparable to the rate in AS when all choices are interior, but their average frequency is higher as there are many more interior choices. In the replication experiment we find a very high rate of demand monotonicity violations and we document that time inconsistent choices are positively associated with classical monotonicity violations that were possible through the experimental interface, suggesting a possible relation between rationalizable choices and time consistency.

We point out the importance of inclusion of demand monotonicity and wealth monotonicity tests in experimental design as diagnostic tests of meaningful economic behavior. Unfortunately, the data does not permit us to go a step further to test our conjectures about the source of these problems. As more studies employing CTBs that also include checks of our monotonicity measures are performed, we would learn more about whether these patterns point to something systematic in subject choices or are merely a result of the particular experimental interface. We believe that further investigation into the origin of the regularities documented in the present study is crucial for an informed interpretation of existing and new experimental results utilizing CTB method and we look forward to exciting insightful work in this field in the near future.

## Footnotes

1 It is often assumed that preferences are time invariant (stable) so that $D_{s}\equiv D$ and $u_{s}\equiv u$ for all $s\ge 0$. For experimental evidence on the validity of this assumption see Halevy (2015).

2 Since if *u* is concave then $c^{1}/c^{2}<u(c^{1})/u\left( c^{2}\right)$ for $c^{1}<c^{2}$.

3 Some form of event separability is still required for incentive compatibility.

4 Recently, Attema et al. (2016) proposed a Direct Method to measure the discount function that relies on ranking of accumulate income, and does not require the adjustment to the curvature of the felicity function.

5 This comparative static treatment was included in the original AS paper, but as will be clear below, we believe that the original paper did not emphasize its importance for evaluating the internal consistency of choices.

6 See Augenblick et al. (2015, p. 1106) and Halevy (2015, p. 350).

7 We thank Andreoni and Sprenger for bringing up this possibility. However, to be consistent with this knife edge case, subjects need to satisfy: (1) $c_{t}^{*}=0$ for all $r>0.25$ and $c_{t+k}^{*}=0$ for all $r<0.25.$ (2) In every choiceset $(t,k')$ such that $k'<k$: $c_{t}^{*}=0$ for all $r\ge 0.25.$ (3) In every choice set $\left( t,k'\right)$ such that $k'>k$: $c_{t+k'}^{*}=0$ for all $r\le 0.25$. (1) follows from linearity and (2–3) follow since the daily rate changes as *k* varies.

8 The discussion in this subsection ignores indifferences since we believe that the evidence is systematic and cannot be accounted for by the knife-edge arguments of linear preferences.

9 Following Harrison et al. (2013) it is important to note that linear felicity function cannot be identified from convex functions using CTB. Under discounted expected utility one can adjust both axes of Fig. 1 using the risk MPL*, only *if the subject is risk seeking in the risk MPL (using the inequality $c^{t}/c^{t+k}>u\left( c^{t}\right) /u\left( c^{t+k}\right)$ for $c_{t}>c_{t+k}$ for convex *u*).

10 AS’ Fig. 4A is similar, but we restrict to subjects who made only corner choices and therefore there is no need to adjust for concavity.

11 If one estimates a quasi-hyperbolic model based on these three CTBs or MPLs, the conclusions do not change. In particular, the present-bias parameter (beta) under both elicitation methods is exactly 1 for 28 out of the 32 subjects.

12 Binary choice is by definition, free of such reference dependence or “attraction effect” in their model.

13 Among the 36 subjects who made only corner choices, we find only one non-monotonic choiceset.

14 If we apply a stricter test for subjects with potentially linear preferences (see footnote 7 for details), it affects only 8 choicesets decreasing the average size to 23.2 tokens.

15 By comparison, the AS measure of wealth monotonicity violation as calculated and reported in footnote 25 of their paper, would equal zero in expectation under the assumption of uniform random choice.

16 AS report 8 demand monotonicity violations for the ($t=7,k=70$) choiceset with an average magnitude of 24.6 tokens; in comparison, we find only 7 violations with an average magnitude of 23.4 tokens in this choiceset. AS appear to have erroneously included additional adjustments for $c_{t+k}$, and correcting for this reduces both the number and magnitude of demand monotonicity violations slightly.

17 When removing data points to restore monotonicity we also consider joint violations of demand and wealth monotonicity.

18 Because the AS (2012a) design has no power to detect violations of GARP, any choices made in a choiceset can be rationalized by a utility function, and by Afriat’s theorem the utility function can be chosen to be increasing in $\left( c_{t},c_{t+k}\right)$. This, however, should not be confused with wealth monotonicity, which is a property of the demand function.

19 For the other subject, one needs to remove a single choice.

20 Only 9 of the 61 subjects made choices fully consistent with monotonicity.

21 200/720 compared to 70/873. Even when excluding choicesets with no interior choices the difference is high: 200/643 = 31.1% compared to 69/440 = 15.7%.

22 We comment on the same for the replication study effort tasks in the next subsection.

23 For example, when $e_{t}=0$ and task rate = 1.5, $e_{t+k}=\lfloor \frac{50-0}{1.5}\rfloor =33$. Here $e_{t}$ is the effort allocation in period $t$ which can vary from 0 to 50.

24 With discount rate $R=1.05263$ and total budget $=60$, all of $(e_{t},e_{t+k})=(40.6,17.26),(40.4,17.47),(40.1,17.78)$ satisfy the budget equation $R*e_{t}+e_{t+k}=60$.

25 Only 3 out of the 5 discount rates used in first effort experiment are presented in the replication study, and hence for the other two we choose the closest approximate. In the original effort data $R=(0.5,0.75,1,1.25,1.5)$ where the budget line is: $e_{t}+R*e_{t+k}=50$ (*R* is called the “task rate” so $R=1/(1+r)$ where r is the discount rate). In the replication experiment $P=(0.666666,0.8,0.90909,0.952381,1,1.05263,1.111111,1.25,1.53846)$ where the budget line is $P*e_{t}+e_{t+k}=60$ (so $P=1+r$), therefore, the relevant values for effort would be $P=\left( 0.66,0.8,1,1.25,1.54\right)$.

26 Because the full $10\times 17$ table is both unwieldy and has a sparsity index of 0.35, we present a condensed version of the table. Clearly, the categorical cutoffs chosen can affect the degree of correlation evident in the condensed table. Nevertheless, it is very clear from the raw data that subjects with moderate to high numbers of classical monotonicity violations are more likely to make dynamically inconsistent choices. Reinforcing this correlation is the fact that classical monotonicity violations, at the subject level, are also correlated between the two decision making periods.

27 This may be related to a finding in Halevy (2015) who finds that time consistency is tightly associated time invariant (stable) choices.

28 In a recent working paper Janssens et al. (2015) test their data, collected in the field using CTB, for violations of demand monotonicity and find that the rate of violations never exceeds 11.5% of choicesets. Their data does not contain the experimental variation required to test for wealth monotonicity, impatience monotonicity or WARP violations.
